# Lifestyle choices and mental health: a longitudinal survey with German and Chinese students

**DOI:** 10.1186/s12889-018-5526-2

**Published:** 2018-05-16

**Authors:** Julia Velten, Angela Bieda, Saskia Scholten, André Wannemüller, Jürgen Margraf

**Affiliations:** Mental Health Research and Treatment Center, Clinical Psychology and Psychotherapy, Ruhr-Universität Bochum, Massenbergstr. 9-13, 44787 Bochum, Germany

**Keywords:** Lifestyle, Positive mental health, Depression, Anxiety, Stress, Physical activity, Body mass index, Alcohol, Smoking, Social rhythm

## Abstract

**Background:**

A healthy lifestyle can be beneficial for one’s mental health. Thus, identifying healthy lifestyle choices that promote psychological well-being and reduce mental problems is useful to prevent mental disorders. The aim of this longitudinal study was to evaluate the predictive values of a broad range of lifestyle choices for positive mental health (PMH) and mental health problems (MHP) in German and Chinese students.

**Method:**

Data were assessed at baseline and at 1-year follow-up. Samples included 2991 German (*M*_age_ = 21.69, *SD* = 4.07) and 12,405 Chinese (*M*_age_ = 20.59, *SD* = 1.58) university students. Lifestyle choices were body mass index, frequency of physical and mental activities, frequency of alcohol consumption, smoking, vegetarian diet, and social rhythm irregularity. PMH and MHP were measured with the Positive Mental Health Scale and a 21-item version of the Depression Anxiety and Stress Scale. The predictive values of lifestyle choices for PMH and MHP at baseline and follow-up were assessed with single-group and multi-group path analyses.

**Results:**

Better mental health (higher PMH and fewer MHP) at baseline was predicted by a lower body mass index, a higher frequency of physical and mental activities, non-smoking, a non-vegetarian diet, and a more regular social rhythm. When controlling for baseline mental health, age, and gender, physical activity was a positive predictor of PMH, smoking was a positive predictor of MHP, and a more irregular social rhythm was a positive predictor of PMH and a negative predictor of MHP at follow-up. The good fit of a multi-group model indicated that most lifestyle choices predict mental health comparably across samples. Some country-specific effects emerged: frequency of alcohol consumption, for example, predicted better mental health in German and poorer mental health in Chinese students.

**Conclusions:**

Our findings underline the importance of healthy lifestyle choices for improved psychological well-being and fewer mental health difficulties. Effects of lifestyle on mental health are comparable in German and Chinese students. Some healthy lifestyle choices (i.e., more frequent physical activity, non-smoking, regular social rhythm) are related to improvements in mental health over a 1-year period.

**Electronic supplementary material:**

The online version of this article (10.1186/s12889-018-5526-2) contains supplementary material, which is available to authorized users.

## Background

Mental health is recognized as a critical component of public health [1]. The need for health promotion, prevention, and treatment programs for mental disorders is one of the primary health challenges of the twenty-first century [2]. The World Health Organization (WHO) describes mental health as a “state of well-being in which every individual realizes his or her own potential, can cope with the normal stresses of life, can work productively and fruitfully, and is able to make a contribution to her or his community” [3]. In line with this definition, mental health researchers increasingly acknowledge that the absence of mental illness does not necessarily imply a state of psychological well-being [4–6]. Mental health problems (MHP) can be defined as the presence of psychopathological symptoms (e.g., depressive mood, excessive anxiety, or compulsive behavior) that indicate mental disorders defined in the classification systems of the American Psychiatric Association [7] or the WHO [8]. Two theoretical approaches are most relevant for positive mental health (PMH), as it is interpreted in this study: From a hedonic perspective, PMH includes positive affect, positive mood, and high life-satisfaction, whereas from an eudaimonic perspective, PMH is an optimal functioning of an individual in everyday life (for more information, please see [4, 9, 10]. Thus, PMH is defined as the presence of general emotional and psychological well-being [11]. For the current study, both hedonic and eudaimonic approaches were taken into account and assessed with the Positive Mental Health Scale [12].

### Lifestyle and health

It is commonly known that leading a healthy life can be beneficial for one’s well-being. But what exactly does a healthy lifestyle entail? According to the WHO, a healthy lifestyle means to engage in regular physical activity, to refrain from smoking, to limit alcohol consumption, to eat healthy food in order to prevent overweight. These behaviors should lead not only to better physical health, but also foster mental well-being [13]. The WHO fact sheet is supported by research data showing that engaging in sports or moderate to rigorous physical activity [14], partaking in cultural or mental activities [15, 16], refraining from smoking [17], practicing moderation in alcohol consumption [18], maintaining a body mass index (BMI) within the range of normal weight [19], and following a healthy diet [20] can have positive health effects and reduce the risk of various somatic diseases, including cancer [21], heart disease [22], or stroke [23]. In addition to the relevance of lifestyle for physical health, findings concerning the importance of healthy lifestyle choices for both PMH and MHP are accumulating, with prospective studies consistently finding a bidirectional relationship between lifestyle and mental health variables [24, 25]. More precisely, studies showed that lifestyle can have a positive effect on symptoms of depression and anxiety [25, 26], life satisfaction [27], and self-perceived general mental health [28–30]. In order to investigate the relevance of lifestyle on mental health we selected seven lifestyle factors that, according to the WHO fact sheet [13] and our own data [31–33], show significant associations to mental health outcomes.

### Which lifestyle choices are beneficial to mental health?

#### Body mass index

Obesity—which describes severe overweight with a BMI higher than 30 [34]—is associated with worse MHP, especially with self-reported symptoms of depression, anxiety, and stress [35]. In a sample of 886 midlife women, higher BMI was associated with symptoms of anxiety and depression and also with lower PMH, measured with a mental health subscale of the Medical Outcomes Study Short-Form 36 (SF-36; [36]), a questionnaire that measures well-being and quality of life [25]. In another population-based study of 7937 adults, higher BMI was associated with higher MHP, namely symptoms of depression, anxiety, and stress, and also predictive of lower PMH measured with the Satisfaction with Life Scale [37], a short measure designed to assess the judgmental component of personal well-being [33]. A meta-analysis of longitudinal studies that assessed the relationship between overweight—a BMI between 25 to 29.99—obesity, and mental health in 58,745 participants showed that both overweight and obesity predicted future symptoms of depression as well as onset of a depressive disorder [38].

#### Physical activity

In a review of prospective studies, physical activity was identified as a protective factor against the risk of developing depression [39]. In patients with chronic medical conditions, exercise training interventions reduced symptoms of anxiety. Compared to non-exercise control conditions, a small reduction in anxiety symptoms was found (*d* = 0.29) [40]. Individuals diagnosed with Major Depression participating in an aerobic exercise intervention showed significant improvements in depression comparable to participants receiving pharmacological treatment [41]. In a meta-analysis of intervention studies in older adults, aerobic exercise (*d* = 0.29) and moderate intensity training (*d* = 0.34) were most beneficial for participants’ psychological well-being [42].

#### Mental or cultural activities

Receptive (e.g., visiting museums or concerts) and creative (e.g., playing an instrument or painting) cultural or mental activities were associated with lower MHP, namely symptoms of anxiety and depression, and higher PMH, namely life satisfaction [33, 43]. In a large longitudinal study including more than 16,000 middle-aged individuals, cultural leisure-time activities were predictive of lower MHP at follow-up 5 years later [44]. Not all studies supported the relevance of mental activities for mental health. In a prospective study with first-year medical students, leisure-time activities such as playing music or being active in a religious community were not predictive of self-reported mental health at follow-up 1 year later [45]. As bivariate correlations and non-significant regression coefficients for cultural activities and mental health were not reported, the findings of this study should be interpreted with caution.

#### Alcohol consumption

The relationship between alcohol consumption and mental health is quite controversial. While some studies identified a nonlinear relationship—with elevated risks for depression and anxiety for abstainers and heavy drinkers as compared to light/moderate drinkers [46]—other studies did not find a meaningful correlation between alcohol consumption and symptoms of MHP [25]. Individuals who identify themselves as alcohol abstainers show higher levels of anxiety and depression in comparison to those who do not report alcohol consumption but do not label themselves abstainers [47]. It is, however, problematic to interpret these findings to support the positive effects of moderate alcohol consumption, as many individuals who abstain from alcohol do so for a reason, such as a history of alcohol abuse or other health issues [47]. To identify the relevance of other confounding variables in the relationship between a facet of PMH, life satisfaction, a series of analyses was conducted using a large population sample in Russia [48]. The u-shaped relationship between alcohol consumption and life satisfaction that was found in men and women, was flattened when sociodemographic factors (e.g., age, gender, and occupational status), smoking, and BMI where included as control variables. In other words, the positive relationship between moderate alcohol consumption and mental health is likely to be influenced by other sociodemographic characteristics or lifestyle factors and not caused by the alcohol consumption itself [48].

#### Smoking

Smoking has been identified as a risk factor for MHP [49, 50]. A meta-analysis of prospective studies with follow-up periods between 7 weeks and 9 years showed that individuals who quit smoking experience a significant decrease in MHP—symptoms of depression, anxiety, and stress—and an increase in PMH—psychological quality of life and positive affect—compared to continuing smokers [51]. In line with these findings, a Dutch study with more than 5000 participants showed that individuals who quit smoking do not experience a loss in life satisfaction, but rather an increase in well-being [52]. During early adolescence, smoking is associated with MPH and especially symptoms of depression [53]. The relationship between mental health and smoking is bidirectional: Young smokers with anxiety and depression are more likely to develop a nicotine addiction in early adulthood [54].

#### Vegetarian diet

Compared to other lifestyle choices, a vegetarian diet has only rarely been investigated in the context of mental health. In a prospective study of more than 9000 young women in Australia, vegetarians and semi-vegetarians (i.e., individuals who excluded red meat, but would eat other meat, poultry, and fish) were more likely to experience mental health problems such as symptoms of depression and anxiety, or sleeplessness [55]. They also reported lower PMH as measured by the SF-36. In a representative study of German adults, individuals who reported a vegetarian diet were more likely to be diagnosed with depressive, anxiety, somatoform, or eating disorders [56]. These findings are in stark contrast to the proposed positive effects of a vegetarian diet on physical health (e.g., [57]). Authors propose that these findings may be caused by psychological traits that influence both eating habits and mental health such as perfectionism. An alternative explanation is based on the finding that mental health difficulties often precede a vegetarian diet. Individuals who experience mental health difficulties may try to modify their behavior in a way that is perceived as healthier or their mental health issues may sensitize them to the suffering of animals [56]. The relevance of a vegetarian diet for PMH and MHP has not been investigated in a prospective study including a variety of other lifestyle choices. As prevalence of a vegetarian diet varies drastically between, for example Asian and European countries [57], the lack of cross-cultural studies including more than one country is another shortcoming in the literature [56].

#### Social rhythm irregularity

The association between MHP and disturbances of circadian rhythms is documented, especially for schizophrenia, bipolar disorder, and depression [58]. Disruptions of the circadian rhythm may trigger or exaggerate manic episodes [59]. There is evidence that the circadian system does also influences one’s capacity for mood regulation [60]. Additionally, an irregular social rhythm, which includes social contacts, are also associated with mood disorders in elderly patients [61]. In one of the first population-based studies investigating rhythm irregularity and mental health, irregular circadian and social rhythm were both associated with more MHP and lower life satisfaction in a German sample [33]. This finding was replicated cross-culturally in representative Russian and Chinese samples [31]. Prospective studies concerning the relevance of social rhythm irregularity on future MHP and PMH are still lacking.

While some longitudinal studies indicate the relevance of lifestyle factors for future MHP, evidence for the prediction of future PMH is much rarer. In addition, most studies focus on one or two lifestyle choices and do not investigate the relative importance of a broad range of behaviors for PMH and MHP. As many lifestyle choices are interrelated—for example, individuals who exercise regularly are less likely to be overweight or obese—those studies do not explain the individual contribution of certain lifestyle choices on mental health outcomes. Lastly, the majority of studies only include U.S. American or European participants. The focus on Western samples precludes cross-cultural conclusions about the relevance of lifestyle choices for PMH and MHP. Germany is an individualistic Western country which has undergone structural changes in the 1990s (specifically the reunification of West and East Germany) [62]. In contrast, China is a collectivistic Asian country [63, 64] in which old values and traditions interact with a rapid economical and technical development (e.g., [65]). Thus, for this study, data from German and Chinese students were analyzed as both countries differ regarding various cultural, historical, social, and geographical conditions.

### The present study

The aim of this study was to overcome these limitations by investigating the impact of seven major lifestyle factors—BMI, physical and mental activities, alcohol consumption, smoking, vegetarianism, and social rhythm irregularity—on concurrent and future PMH and MHP in two large student samples from Germany and China. We predicted that a lower BMI, a higher frequency of physical and mental activities, a lower frequency of alcohol consumption, non-smoking, a non-vegetarian diet, and a more regular social rhythm would be predictive of better mental health at baseline, operationalized as higher PMH and fewer MHP. As longitudinal studies including a variety of lifestyle factors are lacking, no predictions about the relevance of specific lifestyle factors for the prediction of future mental health were made. We expected, however, that mental health at baseline would predict mental health at follow-up.

## Method

### Procedure

The present study was conducted as part of the Bochum optimism and mental health studies (BOOM-studies), which aim to investigate risk and protective factors of mental health in population-based and student samples with cross-sectional and longitudinal assessments across different cultures. Data presented in this study were collected in different student cohorts between 2012 and 2016 in a German (Ruhr-Universität Bochum) and three Chinese universities (Capital Normal University Beijing, Hebei United University, and Nanjing University). Participants in both countries were sent an invitation to the study via email. They received no incentives for participation. Data were assessed through self-administered surveys (i.e., paper-pencil and online surveys in China and an online survey in Germany). Depending on the assessment method, participants gave their informed consent written or online after being informed about anonymity and voluntariness of the survey. Language specific versions of psychometric instruments were administered using the forward-backward-translation method [66]. In case of discrepancies, the procedure was repeated until complete agreement was achieved. All procedures were carried out in accordance with the provisions of the World Medical Association Declaration of Helsinki (2013). The Ethics Committee of the Faculty of Psychology of the Ruhr-Universität Bochum approved the study in total (Reference number 073). Since the data were anonymized from the beginning of data collection, no statement by an ethics committee was required in China. The participating Chinese Universities were, however, informed and acknowledged the approval by the German ethics board. The Chinese sample included students below age 18. Chinese laws grant inscribed university students of all ages the rights to decide for themselves about study-related issues including participation in studies. Thus, no consent to participate was collected from the parents or guardians.

### Participants

In total, 2,991 German and 12,405 Chinese participants had valid data at baseline, which was defined as having data for at least half of lifestyle predictors (see Additional file 1). German participants, *M*_age_ = 21.69, *SD* = 4.07, *range* = 15 to 65, were significantly older than Chinese participants, *M*_age_ = 20.59, *SD* = 1.58, *range* = 15 to 36, *t(*14461*)* = 23.05, *p* < .001, *d* = 0.47. Both samples included more female than male participants (German sample: 58.9% female; Chinese sample: 61.9% female). In the German sample, 50.6% reported being in a committed partnership, while in the Chinese sample 20.8% did so, χ^2^(1) = 1083.37, *p* < .001, *d* = 0.55. Six-hundred and thirty-six German and 8,933 Chinese students had at least one valid value at follow-up.

### Measures

#### Body mass index

Body mass index (BMI) was calculated from weight and height as weight divided by height squared (kg/m^2^). According to the WHO, overweight is a BMI greater than or equal to 25, and obesity is a BMI greater than or equal to 30 [34]. Height and weight were assessed via self-report. Self-reported measurements of height and weight have been found to be very reliable, except for highly obese individuals. For this group, a slight underestimation of weight has been reported [64].

#### Frequency of physical and mental activity

Frequency of physical as well as cultural activities was assessed using items rated on a scale ranging from 0 (*none*) to 3 (*more than 4 times a week*): “Do you exercise/engage yourself in a mental activity regularly? If yes, with what intensity have you done so in the last 12 months?” Participants were provided with examples for physical (i.e., sports and intensive physical work) and mental (i.e., reading, going to the movies/theater, making music) activities. Single-item measures of those activities are characterized by an acceptable reliability and construct validity compared to objective measurement methods [67] and perform at least as well as longer questionnaires [68]. Research has also shown that such items show especially high criterion validity in younger and female participants [68].

#### Alcohol consumption

Frequency of alcohol consumption was assessed using the item “How often do you drink alcohol?” Answer categories were never, once a month, 2 to 4 times a month, 2 to 3 times a week, and 4 times a week and more. While there is an on-going debate about the validity of self-reported alcohol consumption compared to objective data, recent studies conclude that self-report can reliably estimate alcohol consumption in low to moderate drinkers [69]. Such items are regularly used in epidemiological studies on alcohol consumption (e.g., [70]).

#### Smoking

Current smoking was assessed using one item: “Do you smoke regularly?” Answer categories were ‘no’, ‘yes, sometimes’, and ‘yes, regularly’. For the present analyses the two latter categories were combined into ‘yes’, which was coded as 1. ‘No’ was coded as 2.

#### Vegetarian diet

A vegetarian diet pattern was assessed with the item “Do you currently follow a vegetarian diet?”. Answer categories were ‘no’, ‘yes (no meat and no fish)’, ‘yes (no meat)’, ‘vegan’. For this study, the latter three categories were combined to ‘yes’. Similar items were used in previous large-scale studies on eating habits and mental health [56].

#### Social rhythm irregularity

Irregularity of social rhythm was assessed using the Brief Social Rhythm Scale [31] which includes 10 items measuring an individual’s perceived social rhythm regarding sleep, meals, wake-up time, and social contacts on a scale ranging from 1 (*very regularly*) to 6 (*very irregularly*). In the present study, internal consistency was acceptable to good with α = .78 and .90 in the German and Chinese sample, respectively. Validity evidence comes from data indicating that the full scale is related to physical health, consistent with past research on rhythmicity and certain aspects of mental health [31, 33].

#### Positive mental health

The unidimensional Positive Mental Health Scale was used to assess PMH [12]. The PMH-scale is a self-report instrument consisting of nine non-specific judgments and was developed to measure eudaimonic and hedonic aspects of well-being. Targeted at general psychological functioning, subjects were asked to indicate their agreement on a 4-point Likert scale ranging from 0 (*do not agree*) to 3 (*agree*). Example items are “I am often carefree and in good spirits” and “I feel that I am actually well equipped to deal with life and its difficulties”. High internal consistency, good retest- reliability as well as good and discriminant validity were confirmed in a series of six studies comprising samples of students, patients, and general populations [12]. Cronbach’s alpha in the present study was α = .93 in the German and .92 in the Chinese sample.

#### Mental health problems

The negative emotional states of depression, anxiety, and stress—which are subsumed under MHP in this study—were assessed with the Depression Anxiety Stress Scales-21 (DASS-21) [71], a short version of the Depression Anxiety Stress Scales [72]. The DASS-21 consists of 21 items, 7 for each subscale, rated on a 4-point Likert scale ranging from 0 (*did not apply to me at all*) to 3 (*applied to me very much, or most of the time*). Summing across the complete scale yields a total score ranging from 0 to 63. The DASS-21 is a widely used instrument with good psychometric properties [73]. Cronbach’s alpha for the complete scale was α = .92 in the German and α = .95 in the Chinese sample.

### Data analyses

Data were analyzed using SPSS 24 [74] and Mplus 7.4 [75]. Missing data analysis at baseline showed < 1% of missing data concerning most outcome and predictor variables. Three percent of participants had missing values for MHP, 6.1% did not report their age, and 6.9% did not provide valid height and weight estimates to calculate BMI. Missing values were not replaced. For the primary hypotheses, path analysis was used to examine whether the lifestyle factors explained current and predicted future PMH and MHP. To evaluate whether the proposed model would fit the data of German and Chinese students equally well, a multi group path analysis was conducted. To control for differences in sample size, a case-control sample of the Chinese students, matched for age and gender, was randomly drawn and included in this analysis. Cohen’s *d* was calculated as the effect size measure (small effect: *d* ≥ .20, medium effect: *d* ≥ .50, large effect: *d* ≥ .80) [76].

To assess whether the proposed model would fit the data, three fit indices were inspected [77]. The comparative fit index (CFI) compares the hypothesized model’s χ2 with that resulting from the independence model. For an acceptable fit, CFI values above .90 are recommended; a good model fit requires values above .95 [78]. The Root Mean Square Error of Approximation (RMSEA) measures the difference between the reproduced covariance matrix and the population covariance matrix, with values less than .06 reflecting a small approximation error, indicating a good model fit, values between .08 and .10 a mediocre fit and values above .10 a poor model fit [79]. For the standardized root-mean-square residual (SRMR), values smaller than .09 indicated a good fit [79]. Due to the large sample size, the χ2-values were not interpreted.

## Results

### Sample characteristics

Table 1 shows the sample characteristics regarding lifestyle variables and mental health outcomes for Chinese and German participants. In the German sample, no significant differences in baseline PMH, *t(*2977*)* = 0.08, *p* = .934, *d* = 0.00, or MHP, *t(*2622*)* = 0.08, *p* = .935, *d* = 0.00, emerged between baseline-only and other participants, meaning that participation at follow-up was not associated with baseline mental health. In the Chinese sample, baseline-only participants were comparable concerning baseline PMH, *t(*12737*)* = − 1.59, *p* = .111, *d* = 0.03, but showed slightly higher levels of baseline MHP, *t(*12304*)* = 2.21(12304), *p* = .027, *d* = 0.04, than Chinese students who participated at both assessment points. This group difference, however, was very small. For descriptive purposes, Table 2 shows non-parametric bivariate correlations between all predictor and outcome variables.

**Table 1:** - Descriptive values of lifestyle factors and mental health at baseline for the German and Chinese samples

	German students			Chinese students					
	*n*	*M*	*SD*	*n*	*M*	*SD*	*t (df)*	*p*	*d*
Lifestyle factors									
Body mass index	2981	22.88	3.87	11350	20.49	2.66	39.29 (14461)	< .001	0.65
Physical activity (*range*: 0 to 4)	2991	2.24	1.19	12405	2.38	1.05	−6.36 (15394)	< .001	0.10
Mental activity (*range*: 0 to 4)	2991	2.72	1.10	12402	3.05	1.09	−14.62 (15391)	< .001	0.24
Alcohol frequency (*range*: 0 to 4)	2991	1.43	1.00	12371	0.65	.89	42.12 (15360)	< .001	0.68
Social rhythm irregularity (*range*: 10 to 60)	2991	29.58	9.01	12338	26.81	9.33	14.71 (15327)	< .001	0.24
	*n*	%		*n*	%		χ^2^	*p*	*d*
Smoking (% yes)	608	20.3		1355	10.9		191.13 (1)	< .001	0.22
Vegetarian diet (% yes)	482	16.1		2734	22.2		55.45 (1)	< .001	0.12
	*n*	*M*	*SD*	*n*	*M*	*SD*	*t (df)*	*p*	*d*
Mental health at baseline									
Positive mental health (*range*: 0 to 27)	2979	18.13	5.46	12375	19.90	5.21	−16.52 (15352)	< .001	0.27
Mental health problems (*range*: 0 to 63)	2624	15.17	10.98	12306	9.50	10.14	25.65 (14928)	< .001	0.42
	*n*	*M*	*SD*	*n*	*M*	*SD*	*t (df)*	*p*	*d*
Mental health at follow-up									
Positive mental health (*range*: 0 to 27)	632	18.23	5.58	8920	20.15	5.30	−8.79 (9550)	< .001	0.18
Mental health problems (*range*: 0 to 63)	600	14.16	10.53	8834	9.39	10.96	10.32 (9432)	< .001	0.21

**Table 2:** - Nonparametric bivariate correlations (Kendal’s tau) between outcome variables at baseline and follow-up and lifestyle predictors at baseline (German students: Top-right, Chinese students: Bottom-left)

		1	2	3	4	5	6	7	8	9	10	11	12	13
PMH^a^ Baseline	1	1	.52^***^	−.45^***^	−.32^***^	−.06^***^	−.07^***^	−.02	.15^***^	.06^***^	.03^*^	−.08^***^	−.06^***^	−.21^***^
PMH Follow-up	2	.33^***^	1	−.36^***^	−.45^***^	−.06	−.05	−.01	.11^***^	.03	.03	−.04	−.02	−.19^***^
MHP^b^ Baseline	3	−.42^***^	−.24^***^	1	.44^***^	.09^***^	.04^**^	.01	−.11^***^	−.08^***^	−.02	.10^***^	.07^***^	.22^***^
MHP Follow-up	4	−.22^***^	−.41^***^	.29^***^	1	.10^***^	.01	−.01	−.08^***^	−.04	−.09^***^	.03	.02	.18^***^
Gender	5	.00	.02^*^	−.02^*^	−.09^***^	1	−.02	−.21^***^	−.08^***^	.02	−.10^***^	−.01	.13^***^	−.02
Age	6	.02^**^	.05^***^	−.02^**^	−.03^***^	−.02^**^	1	.14^***^	−.08^***^	−.01	.04^**^	.11^***^	.05^**^	.08^***^
Body mass index	7	.01^*^	.01	.00	.00	−.20^***^	.03^***^	1	−.02	−.01	.03^*^	.05^**^	−.06^***^	.05^***^
Physical activity	8	.13^***^	.09^***^	−.11^***^	−.06^***^	−.17^***^	−.07^***^	.07^***^	1	−.01	.05^**^	−.12^***^	−.02	−.13^***^
Mental activity	9	.12^***^	.09^***^	−.11^***^	−.08^***^	−.02^**^	−.03^***^	.02^**^	.19^***^	1	.05^**^	−.03	.07^***^	−.05^***^
Alcohol frequency	10	−.03^***^	−.04^***^	.07^***^	.09^***^	−.39^***^	−.01	.08^***^	.10^***^	−.01	1	.23^***^	.03	.04^**^
Smoking (no/yes)	11	−.05^***^	−.04^***^	.08^***^	.11^***^	−.31^***^	.00	.05^***^	.06^***^	−.06^***^	.38^***^	1	.06^**^	.12^***^
Vegetarian diet (no/yes)	12	−.01	−.03^**^	.04^***^	.07^***^	−.09^***^	−.02^*^	.00	.04^***^	−.07^***^	.07^***^	.13^***^	1	.06^***^
Irregular social rhythm	13	−.26^***^	−.17^***^	.28^***^	.16^***^	−.01	−.07^***^	−.03^***^	−.16^***^	−.13^***^	.08^***^	.07^***^	.02^**^	1

^*^ *p* < .05; ^**^ *p* < .01; ^***^ *p* < .001

^a^positive mental health; ^b^mental health problems

### Lifestyle choices and mental health in German students

PMH and MHP were negatively correlated at baseline, *r* = −.55 (2960), *p* < .001, and follow-up, *r* = −.50 (2960), *p* < .001. Explained outcome variance by lifestyle factors at baseline was 13.2% for MHP, and 13.4% for PMH. Baseline lifestyle explained 10.8% of variance in PMH, and 10.5% of variance in MHP. Table 3 shows the results of the regression paths of the path analysis for the prediction of current and future mental health by lifestyle choices in German students.

**Table 3:** - Path analysis for the prediction of positive mental health and mental health problems by lifestyle choices in German students

Outcome	Positive mental health (PMH)					Mental health problems (MHP)				
Fixed effects	β	SE (β)	*t*	*p*	*d*	β	SE (β)	*t*	*p*	*d*
Cross-sectional analysis										
Gender (male/female)	−.07	0.02	−3.85	< .001	0.15	.11	0.02	6.05	< .001	0.23
Age	−.02	0.02	−1.27	.204	0.05	−.03	0.02	−1.52	.128	0.06
Body mass index	−.04	0.02	−2.13	.033	0.08	.05	0.02	2.39	.017	0.09
Physical activity	.13	0.02	7.64	< .001	0.29	−.07	0.02	−3.56	< .001	0.14
Mental activity	.08	0.02	4.43	< .001	0.17	−.09	0.02	−5.24	< .001	0.20
Alcohol frequency	.04	0.02	2.44	.015	0.09	−.02	0.02	−1.16	.245	0.04
Smoking (no/yes)	−.05	0.02	−2.65	.008	0.10	.08	0.02	4.24	< .001	0.16
Vegetarian diet (no/yes)	−.05	0.02	−2.88	.004	0.11	.07	0.02	3.64	< .001	0.14
Social rhythm irregularity	−.26	0.02	−15.33	< .001	0.59	.28	0.02	15.50	< .001	0.60
Longitudinal analysis										
Baseline positive mental health (PMH)	.55	0.03	16.61	< .001	0.64	−.09	0.04	−2.19	.028	0.08
Baseline mental health problems (MHP)	−.12	0.04	−3.22	.001	0.12	.52	0.04	14.02	< .001	0.54
Gender (male/female)	−.05	0.03	−1.51	.130	0.06	.08	0.03	2.45	.014	0.09
Age	−.02	0.03	−0.56	.573	0.02	.00	0.03	−0.05	.963	0.00
Body mass index	−.03	0.03	−1.11	.265	0.04	.03	0.03	0.77	.442	0.03
Physical activity	.03	0.03	0.82	.414	0.03	.03	0.03	0.76	.447	0.03
Mental activity	−.03	0.03	−1.15	.251	0.04	.00	0.03	−0.04	.970	0.00
Alcohol frequency	−.02	0.03	−0.51	.610	0.02	−.02	0.03	−0.70	.481	0.03
Smoking (no/yes)	.00	0.03	0.11	.910	0.00	.03	0.03	1.01	.311	0.04
Vegetarian diet (no/yes)	.05	0.03	1.62	.105	0.06	−.05	0.03	−1.60	.111	0.06
Social rhythm irregularity	−.09	0.03	−2.72	.007	0.10	.08	0.04	2.19	.028	0.08

#### Positive mental health

Female gender, a higher body mass index, smoking, vegetarian diet, and social rhythm irregularity were predictive of lower PMH at baseline. Higher frequency of physical activity, mental activity, and alcohol consumption were positive predictors of baseline PMH. Effects were mostly small. Somewhat larger effects were found for physical activity (*d* = 0.29) and social rhythm irregularity (*d* = 0.59). PMH and MHP were both predictive of PMH at follow-up. In addition, only social rhythm irregularity was a negative predictor of future PMH.

#### Mental health problems

Male gender, a higher body mass index, smoking, a vegetarian diet, and an irregular social rhythm were positive, physical and mental activity were negative predictors of baseline MHP. Effects were small, except for social rhythm irregularity which had a medium-sized effect (*d* = 0.68). PMH and MHP were both predictive of MHP at follow-up. In addition, being female and having a more irregular social rhythm at baseline were positive predictors of future MHP.

### Lifestyle and mental health in Chinese students

PMH and MHP were negatively correlated at baseline*, r* = −.39 (10803), *p* < .001, and follow-up, *r* = −.36 (10803), *p* < .001. Explained outcome variance by lifestyle factors at baseline was 12.8% for PMH, and 16.1% for MHP. Baseline lifestyle explained 6.4% of variance in PMH, and 8.1% of variance in MHP. Table 4 shows the results of the regression paths of the path analysis for the prediction of current and future mental health in Chinese students.

**Table 4:** - Path analysis for the prediction of positive mental health and mental health problems by lifestyle choices in Chinese students

Outcome	Positive mental health (PMH)					Mental health problems (MHP)				
Fixed effects	β	SE (β)	*t*	*p*	*d*	β	SE (β)	*t*	*p*	*d*
Cross-sectional analysis										
Gender (male/female)	.01	0.01	0.70	.487	0.01	−.01	0.01	−1.08	.279	0.02
Age	.02	0.01	2.36	.018	0.05	−.03	0.01	−2.81	.005	0.05
Body mass index	.01	0.01	0.97	.332	0.01	.03	0.01	3.42	.001	0.07
Physical activity	.09	0.01	9.40	< .001	0.18	−.04	0.01	−4.62	< .001	0.09
Mental activity	.07	0.01	7.05	< .001	0.14	−.09	0.01	−9.77	< .001	0.19
Alcohol frequency	−.04	0.01	−4.12	< .001	0.08	.10	0.01	9.28	< .001	0.18
Smoking (no/yes)	−.03	0.01	−2.85	.004	0.05	.06	0.01	5.69	< .001	0.11
Vegetarian diet (no/yes)	.00	0.01	0.07	.943	0.00	.06	0.01	6.68	< .001	0.13
Social rhythm irregularity	−.29	0.01	−32.32	.000	0.62	.31	0.01	35.12	< .001	0.68
Longitudinal analysis										
Baseline positive mental health (PMH)	.35	0.01	31.79	< .001	0.61	−.12	0.01	−10.18	< .001	0.20
Baseline mental health problems (MHP)	−.08	0.01	−7.17	< .001	0.14	.23	0.01	19.36	< .001	0.37
Gender (male/female)	.03	0.01	3.03	.002	0.06	−.12	0.01	−10.18	< .001	0.20
Age	.06	0.01	5.58	< .001	0.11	−.02	0.01	−2.17	.030	0.04
Body mass index	−.01	0.01	−0.99	.321	0.02	−.01	0.01	−0.83	.406	0.02
Physical activity	.04	0.01	3.27	.001	0.06	−.01	0.01	−0.91	.365	0.02
Mental activity	.03	0.01	2.82	.005	0.05	−.04	0.01	−3.74	< .001	0.07
Alcohol frequency	.00	0.01	−0.03	.975	0.00	.03	0.01	2.15	.031	0.04
Smoking (no/yes)	.00	0.01	0.27	.784	0.01	.06	0.01	5.05	< .001	0.10
Vegetarian diet (no/yes)	−.02	0.01	−2.28	.022	0.04	.06	0.01	5.65	< .001	0.11
Social rhythm irregularity	−.05	0.01	−4.73	< .001	0.09	.03	0.01	2.66	.008	0.05

#### Positive mental health

At baseline, a higher frequency of alcohol consumption, smoking, and a higher social rhythm irregularity were predictive of lower PMH. Being older and reporting a higher frequency of physical and mental activities were positive predictors of PMH. Effects were mostly small. A medium effect was found for social rhythm irregularity (*d* = 0.62). PMH and MHP were both predictive of PMH at follow-up. In addition, male gender, older age, and higher frequency of physical and mental activities were positive predictors of future PMH. Vegetarian diet and a more irregular social rhythm were negative predictors of future PMH. All effects of lifestyle factors on future PMH were small.

#### Mental health problems

At baseline, female gender, a higher body mass index, more frequent alcohol consumption, smoking, a vegetarian diet, and an irregular social rhythm were positive, frequency of physical and mental activities as well as higher age were negative predictors of MHP. Effects were small, except for social rhythm irregularity which had a medium-sized effect (*d* = 0.68). PMH and MHP were both predictive of MHP at follow-up. In addition, younger and female participants, those with fewer mental activities, more frequent alcohol consumption, smokers, vegetarians, and those having a more irregular social rhythm reported higher MHP at follow-up. All effects of lifestyle factors on future MHP were small.

### Lifestyle and mental health across samples

To evaluate the impact of lifestyle choices for PMH and MHP across samples, a multi-group path analysis was conducted including all German participants (*n* = 2800) as well as a randomly selected sample of Chinese students (*n* = 2745) that was matched for age and gender (see Additional file 2). While correlations between PMH and MHP, intercepts, and residual variances were allowed to differ, all regression paths were set equal between countries. Different indices supported a good fit of this model (RMSEA = .043, CFI = .955, SRMR = .048). In German and Chinese students, explained outcome variance by lifestyle factors at baseline was 12.5% and 11.2% for PMH, and 11.9% and 13.1% for MHP, respectively. Lifestyle at baseline explained 5.8% and 6.0% of variance in future PMH, and 7.6% and 8.5% of variance in future MHP in German and Chinese students. Table 5 shows the results of the regression paths of the path analysis for the prediction of current and future mental health in our multi-group model of German and Chinese students.

**Table 5:** - Multi-group path analysis for the prediction of positive mental health and mental health problems by lifestyle factors in German and Chinese students (matched data set)

Outcome	Positive mental health (PMH)					Mental health problems (MHP)				
Fixed effects	β	SE (β)	*t*	*p*	*d*	β	SE (β)	*t*	*p*	*d*
Cross-sectional analysis										
Gender (male/female)	−.04	0.01	−2.71	.007	0.07	.04	0.01	3.29	.001	0.09
Age	.00	0.02	−0.11	.916	0.00	−.05	0.02	−3.19	.001	0.09
Body mass index	−.03	0.02	−2.15	.031	0.06	.06	0.02	3.83	< .001	0.10
Physical activity	.12	0.01	8.45	< .001	0.23	−.05	0.01	−3.25	.001	0.09
Mental activity	.07	0.01	4.92	< .001	0.13	−.06	0.01	−4.87	< .001	0.13
Alcohol frequency	.00	0.02	0.03	.974	0.00	.03	0.02	2.37	.018	0.06
Smoking (no/yes)	−.06	0.02	−4.13	< .001	0.11	.10	0.02	6.43	< .001	0.17
Vegetarian diet (no/yes)	−.03	0.01	−2.31	.021	0.06	.07	0.01	6.15	< .001	0.16
Social rhythm irregularity	−.27	0.01	−20.98	< .001	0.56	.27	0.01	21.08	< .001	0.56
Longitudinal analysis										
Positive mental health (PMH)	.42	0.02	19.99	< .001	0.53	−.11	0.02	−5.22	< .001	0.14
Mental health problems (MHP)	−.14	0.02	−6.18	< .001	0.17	.39	0.02	17.49	< .001	0.48
Gender	.00	0.02	0.16	.870	0.00	−.06	0.02	−2.75	.006	0.07
Age	.04	0.02	1.84	.065	0.05	−.05	0.02	−2.22	.027	0.06
Body mass index	−.04	0.02	−1.81	.070	0.05	−.03	0.02	−1.10	.273	0.03
Physical activity	.04	0.02	1.97	.048	0.05	−.02	0.02	−0.96	.335	0.03
Mental activity	−.01	0.02	−0.49	.624	0.01	−.01	0.02	−0.24	.808	0.01
Alcohol frequency	−.04	0.02	−1.79	.074	0.05	.03	0.02	1.49	.136	0.04
Smoking (no/yes)	.02	0.02	0.83	.406	0.02	.09	0.02	3.61	< .001	0.10
Vegetarian diet (no/yes)	.00	0.02	0.24	.810	0.01	.03	0.02	1.63	.103	0.04
Social rhythm irregularity	−.05	0.02	−2.44	.015	0.07	.06	0.02	2.81	.005	0.08

#### Positive mental health

At baseline, female gender, a higher body mass index, smoking, a vegetarian diet, and a more irregular social rhythm were predictive of *lower* PMH. In addition, higher frequencies of physical and mental activities were positive predictors of PMH. Effects were small, except for social rhythm irregularity which had a medium effect on PMH (*d* = 0.56). PMH was a positive and MHP were a negative predictor of PMH at follow-up. In addition, reporting higher frequency of physical activities was a positive predictor, while a more irregular social rhythm was a negative predictor of future PMH. All effects of lifestyle factors on future PMH were small.

#### Mental health problems

At baseline, female gender, higher body mass index, more frequent alcohol consumption, smoking, a vegetarian diet, and an irregular social rhythm were positive, older age and a higher frequency of physical and mental activities were negative predictors of MHP. Effects were small, except for social rhythm irregularity which had a medium-sized effect on MHP (*d* = 0.56). PMH was a negative and MHP were a positive predictor of MHP at follow-up. In addition, female gender, younger age, smoking, and having a more irregular social rhythm at baseline were positive predictors of future MHP. All effects of lifestyle factors on future MHP were small.

## Discussion

The primary objective of this study was to evaluate the predictive value of a broad range of lifestyle choices for PMH and MHP in a cross-cultural study using two longitudinal student samples from Germany and China. In both samples, the lifestyle factors under investigation explained a substantial amount of variance in mental health outcomes at baseline and at follow-up. In a multi-group model including samples of German and Chinese students that were matched for gender and age, some lifestyle choices—physical activities, smoking, and social rhythm irregularity—were predictive of future PMH and/or MHP even when controlling for age, gender, and baseline mental health. A good fit of this multi-group model indicated that, overall, the impact of lifestyle on PMH and MHP was comparable across countries. These findings suggest that choosing healthier lifestyle behaviors can increase psychological well-being and reduce symptoms of depression, anxiety, and stress. We found, however, some differences in lifestyle between German and Chinese students as well as a differential impact of certain lifestyle choices on the mental health of the two groups. In the following section, we will first describe differences in lifestyle and mental health between the two student samples and proceed to discuss which hypotheses were supported cross-culturally or within one of our samples.

### Lifestyle choices in German and Chinese students

German and Chinese students lead different lifestyles. Medium to large group differences were found for BMI and alcohol consumption, with higher values for German compared to Chinese students. Despite increasing levels of overweight and obesity in Asian children, adolescents, and adults, high BMI [80] are still much more prevalent in Europe than in Asia [81]. In addition, Germany is among the countries with the highest alcohol consumption rates worldwide [82]. China has undergone substantial social and economic changes with increasing urbanization, changes to traditional family structure, and developments towards a free market which have been accompanied by an increase in alcohol consumption [83] and changed dietary habits shifting away from high-carbohydrate foods toward high-fat, high-energy density foods [84]. Our findings, however, suggest that on an absolute level, German students still surpass their Chinese counterparts with respect to these two lifestyle factors.

The remaining differences, even though statistically significant, were minimal. Chinese students reported engaging in mental and physical activities more frequently, as well as having a more regular social rhythm. Fewer Chinese students were smokers. Overall, Chinese students lead a somewhat healthier lifestyle than German students except for more Chinese participants indicating a vegetarian diet. It may, however, be an oversimplification to devaluate a meat-free diet as an unhealthy lifestyle choice as positive effects of a vegetarian diet on physical health are well documented [85] and research on the relationship between vegetarianism and mental health is still in its infancy (see the following section for more information).

### Mental health in German and Chinese students

Chinese students showed higher levels of PMH at baseline than their German counterparts (*d* = 0.27). When comparing these findings to a previous study that included three population-based samples in Germany, Russia, and the United States, both student samples scored significantly lower than all of these samples [31]. This finding can not only be explained by the small, but significant correlation between age and PMH. In other words, these findings support the notion that college students report higher mental disorder prevalence rates than the general population [86]. Regarding birth cohorts, psychopathology among college students increased [87].

With respect to MHP at baseline, differences between German and Chinese students were even larger (*d* = 0.42). Again, German students showed values that were substantially higher than those reported in a representative study in Germany [31]. Surprisingly, Chinese students reported much fewer MHP than expected based on previous studies using the DASS-21 in Asian samples [88]. A potential explanation could be the face-to-face assessment method used in our Chinese sample, which might lead to more socially desirable responding and lower levels of MHP compared to online surveys [89].

### Lifestyle choices and mental health across countries

Lifestyle choices assessed in this study explained a small to medium amount of PMH and MHP variance in both German and Chinese students at baseline. Effect sizes were comparable to those found in another cross-sectional study that investigated a similar set of predictors of life satisfaction and MHP in a population-based sample of German adults [33]. Lifestyle choices explained a small amount of variance in PMH and MHP at follow-up even when controlling for baseline mental health. Thus, other variables, that were not included in the study such as socioeconomic factors, could explain more variance. Next to lifestyle choices that are manifested in behaviors, internal factors, like personality (e.g. neuroticism or extraversion) [90], positive factors (e.g., resilience or social support) [91], and cognitive as well as emotional processes (e.g. rumination or other emotion-regulation strategies) [92, 93] also influence PMH and MHP.

#### Body mass index

The predictive value of BMI for PMH differed between samples: While a higher BMI was indicative of more MHP in both countries, only in Germany it was associated with lower PMH as well. While all effect sizes were small, the predictive value of BMI for mental health at both time-points was somewhat higher in German than in Chinese students. These findings might be related to the, on average, higher level of BMI and its greater variance in Germany. A substantial number of Chinese students were underweight (BMI < 18.5), (21.7% vs. 6.4% in the German sample), which is a known risk factor for MHP [94]. To reduce complexity, a quadratic polynomial of BMI was not added to our analyses. In order to further investigate the impact of underweight, normal weight, and overweight on PMH and MHP in (Chinese) college students future studies should include different BMI variables (i.e., linear and quadratic terms).

#### Physical and mental activities

As hypothesized, both physical and mental activities were independently predictive of higher PMH and fewer MHP at baseline. Both variables were also predictive of future PMH in Chinese students above and beyond baseline mental health. In our multi-group model, physical activity was associated with increases in PMH from baseline to follow-up. Our findings underline the importance of exercise, sports, and cultural or mental leisure-time activities [33, 39, 43] for psychological well-being as well as for the prevention of mental disorders. Effects were small but compared to other lifestyle choices, the impact of both variables on PMH and MHP was the second and third largest in size. These findings imply that lifestyle interventions that aim to increase physical activities could not only strengthen students’ physical health (i.e., reduce overweight) [14], but may also improve mental health outcomes [95].

#### Alcohol consumption

Although previous evidence concerning alcohol consumption and mental health was mixed [25, 46], we assumed that more frequent alcohol consumption would be associated with more MHP and lower PMH in our student samples. Our hypothesis was supported only in the Chinese sample. In German students, more frequent drinking was indeed predictive of higher PMH and was not a predictor of MHP. In other words, German students who reported more frequent drinking, showed greater psychological well-being. Previous studies indicated that positive associations between alcohol intake and mental health are moderated by other confounding variables [48]. Thus, it seems unlikely that the alcohol intake itself is responsible for improved mental health. A possible explanation might be that German students who consume alcohol on a regular basis, exhibit other social or trait characteristics (e.g., higher socioeconomic status, more social support, more openness to experience) that are related to higher PMH and fewer MHP.

#### Smoking

Our assumptions concerning smoking being associated with more MHP and lower PMH were supported. Using longitudinal data, smoking was a positive predictor of MHP in Chinese students as well as our multi-group model. In other words, students who were smokers at baseline, reported an increase in symptoms of depression, anxiety, and stress from baseline to follow-up. In both samples and across both assessment points, smoking was more strongly associated with MHP than with PMH. Further dissemination of smoking cessation programs might help more college students to cease smoking and thereby not only improve their physical, but also their mental health [96].

#### Vegetarian diet

As hypothesized, students who reported a vegetarian diet had lower PMH and more MHP compared to other participants. This finding is in line with a previous study in German adults [56] and is especially interesting as rates of vegetarians were high in both of our student samples (16% in German and 22% in Chinese students). Although effects were small, in Chinese students a vegetarian diet was also a significant predictor of future PMH and MHP when controlling for other lifestyle choices, age, gender, and baseline mental health. This finding contradicts the proposition that many individuals become vegetarians in order to cope with ongoing mental health issues [56]. A possible explanation is that—similar to the positive effect of alcohol consumption on German students’ mental health—a vegetarian diet might be related to other factors not assessed in this study (e.g., a lower socioeconomic status or worrying and rumination about animal suffering) which mediate this relationship.

#### Irregular social rhythm

As expected, a more irregular social rhythm—not going to bed or eating meals at a similar time every day—was predictive of lower PMH and more MHP in both samples. Compared to other lifestyle choices, social rhythm irregularity showed the strongest associations with mental health across samples (*d* > 0.55). In line with the social zeitgeber theory (*Zeitgeber* is German for time–giver), disruptions in time-cues that trigger the body’s patterns of biological and social behavior can lead to increased MHP [97, 98]. Our findings suggest that more irregular social rhythms—that may be typical in college students who must deal with varying course schedules, periods of intensive learning, part-time jobs, and non-lecture periods—can be detrimental to students’ mental health across countries.

### Implications

This study supports the cross-cultural relevance of lifestyle for students’ mental health by suggesting that some lifestyle choices may be more relevant for PMH and MHP than others. As a more regular social rhythm was predictive of future mental health in Chinese and German students, more precise measures including ambulatory assessment methods should be applied to assess this factor with more precision and to minimize recall biases. Use of a more precise instrument, like the Social Rhythm Metric [99], can help distinguish between frequency and regularity of social activities. The same is true for mental/cultural activities which were also predictive of future mental health in Chinese students. As most studies into receptive (e.g., reading, listening to music) and creative (i.e., playing an instrument) leisure time activities have been conducted in Western countries (i.e., Norway) [43], more studies are needed to identify which cultural/mental activities are accessible and beneficial to mental well-being in Asian students. For many individuals, young-adulthood is a phase of transition which can be characterized by the pursuit of educational opportunities and employment prospects as well as development of personal relationships which can foster personal growth but may also lead to stress that precipitates the onset or recurrence of MHP [100]. From a public health perspective, it may be promising for campuses to promote low-barrier lifestyle interventions (e.g., [97]) to improve not only students’ physical, but also mental health. Such programs or interventions may be especially useful to reach the large number of students with MHP who do not seek out services that directly address mental health issues (i.e., psychological counselling centers) [101].

### Limitations

Some limitations reduce the validity and reliability of our findings. The use of student samples precludes generalization of our findings to other populations (i.e., older individuals or those with lower education levels). While baseline mental health of individuals who did not to participate at follow-up was similar to individuals who have partaken in both assessment points, it might be possible that individuals whose mental health worsened over the course of the study period were less inclined to participate again. Lifestyle choices explained only a small to medium amount of variance in MHP and PMH. Thus, other factors not included in this study might also be relevant for students’ mental health. Although we aimed to include a broad range of lifestyle factors, some aspects such as religious activities, game playing, or travel were not assessed [102]. Furthermore, we did not ask the participants whether they carried out their activities alone or with others, a factor that impacts the relationship between lifestyle choices and mental health, especially in men [44]. While our outcome variables as well as social rhythm irregularity were assessed with carefully constructed psychometric instruments, body mass index, physical and mental activities, alcohol consumption, smoking, vegetarian diet, and body mass index were assessed with only one item each. In a study combining daily diary entries as well as retrospective single-item self-reports of different behaviors, students’ alcohol use was reliably assessed via single-item self-report. Similar single-item measures have been used in several previous studies [67], and have exhibited sufficient reliability and validity; memory biases and socially desirable responding may, however, have had an additional effect on these items.

## Conclusion

Investigating a broad range of lifestyle choices in German and Chinese students showed that lifestyle has a significant impact on students’ psychological, emotional, and social well-being as well as on symptoms of mental health difficulties. Across samples, a lower body mass index, more frequent physical and mental activities, non-smoking, a non-vegetarian diet, and a more regular social rhythm were associated with better mental health. While some differences between German and Chinese students emerged, a multi-group model showed that most lifestyle factors predict students’ mental health similarly across countries. As some lifestyle choices—physical activity, non-smoking, and a more regular social rhythm—predicted the state of future mental health when controlling for age, gender, and baseline mental health, interventions promoting lifestyle changes in students may be effective in improving students’ mental health.

## Additional files

Additional file 1: Complete_dataset. Complete data set of Chinese and German students. (XLS 5085 kb)

Additional file 2: Matched_dataset. Complete data set of Chinese and German students. (XLS 1756 kb)

## Abbreviations

BMI: Body mass index
CFI: Comparative Fit Index
DASS-21: Depression Anxiety Stress Scales 21
MHP: Mental health problems
PMH: Positive mental health
RMSEA: Root Mean Square Error of Approximation
SRMR: Standardized Root-Mean-Square Residual
